# Predictive models of suicidal ideation risk in perinatal-stage women based on sociodemographic and clinical data

**DOI:** 10.3389/frai.2026.1774453

**Published:** 2026-05-13

**Authors:** José Manuel Martínez-Ramírez, Rocío Adriana Peinado-Molina, Antonio Hernández-Martínez, Juan Miguel Martínez-Galiano

**Affiliations:** 1 Department of Computer Science, University of Jaen, Jaén, Spain; 2 Department of Nursing, University of Jaen, Jaén, Spain; 3 Consortium for Biomedical Research in Epidemiology and Public Health (CIBERESP), Madrid, Spain; 4 Department of Nursing, Physiotherapy and Occupational Therapy, Faculty of Nursing, University of Castilla-La Mancha, Ciudad Real, Castilla la Mancha, Spain

**Keywords:** artificial intelligence, explainable AI, perinatal suicide, suicidal ideation, suicide risk prediction

## Abstract

**Introduction:**

Suicidal ideation in women during the perinatal period has become a growing public health problem, with a prevalence ranging from 8 to 19%. Its etiology is multifactorial and carries additional consequences for both the newborn and the woman beyond death itself.

**Aim:**

This study aims to predict the risk of suicidal ideation in perinatal women by using artificial intelligence models based on sociodemographic and clinical data.

**Methods:**

An analytical observational study was conducted with a sample of 908 Spanish women during the perinatal period, collecting relevant data. To predict the risk of suicidal ideation, five machine learning models (OneR, JRIP, FURIA, J48 and Random Forest) were employed, as they provide rules or trees that can be easily followed to gather additional information. The metrics used to evaluate the performance of the models included accuracy, precision, recall and F1-score.

**Results:**

The models show an accuracy of around 60% in most cases. The model that performs the worst is OneR, with an accuracy of less than 50%. The Random Forest model stood out for its higher accuracy. The metrics of this model (Random Forest) were Accuracy (%) 0.639 ± 0.05, Precision: 0.634, Recall: 0.634 F1:0.634 and AUPRC: 0.632. Factors identified as predictors of suicidal ideation risk included low birth weight, history of mental health problems, problems of intimate partner violence, low income and smoking.

**Conclusion:**

In conclusion, predictive models based on sociodemographic data and clinical variables show a moderate ability to predict suicidal ideation risk in perinatal women.

## 1 Introduction

The risk of suicidal ideation in women during pregnancy, labor and post-partum is steadily increasing. Different studies claim that the prevalence lies between 8% and 19% (Dudeney et al., 2023; Martínez-Vázquez et al., 2024). This can be extrapolated to the general population, and the World Health Organization (WHO) has been attempting to increase awareness about this particular problem since 2014 (World Health Organization, 2014).

The cause of suicide is multifactorial, although different factors associated with suicidal ideation in the perinatal stage have been identified: sociodemographic factors such as marital status or level of education, clinical-assistance factors such as a history of mental pathology or the treatment received during labor and delivery (World Health Organization, 2014; Martínez-Galiano et al., 2025), or lifestyle factors such as smoking or use of toxic substances (Gelabert et al., 2024; Zivin et al., 2024). Suicide in itself bears further consequences to the newborn and the woman beyond that of the woman’s own death (Shigemi et al., 2021; Legazpi et al., 2022).

In routine pregnancy control, there is no measure recommended either by scientific societies, nor by the health administration to address, identify or prevent the problem, despite its magnitude and consequences. Innovative approaches are urgently needed to improve the detection of suicidal ideation at the perinatal stage (Gelaye et al., 2016). In addition to the awareness of psychological problems and the different mental pathologies during the perinatal period, it is necessary to promote the development of resources that address the development of resources that address the subject (Vacheron et al., 2022).

Given the limitations of current approaches, there is a growing need to enable early and accurate predictions of the risk of suicidal ideation to facilitate timely intervention. Data science has seen a quick development in the latest years, and can be applied to a wide variety of disciplines, including medicine.

Although these tools can often provide accurate results, it is necessary that the systems also can determine why each prediction occurs, so that specialists who make use of them can additionally learn new patterns and causes of higher suicidal ideation risks, aiding in further researches. For instance, Deep Learning (DL) (Goodfellow et al., 2016) models offer extreme accuracy, but their results cannot be explained or justified easily. For this reason, such black box models will be ignored, and only models that can justify their results by offering either rules or trees (explainable AI), which in turn can be Trustworthy Artificial Intelligence (TAI) (Rodríguez Cuadros, 2021), will be used. By gathering enough data, a classification problem can be defined in a way that these models may attempt to extract information from data to predict whether someone has a higher suicidal ideation risk (Hastie et al., 2009). The main purpose of this model is to predict whether a woman has risk of having thoughts about committing suicide based on sociodemographic and clinical data in order to offer relevant assistance if necessary.

## 2 Materials and methods

A study was carried out based on a survey of women in the perinatal stage (pregnant or postpartum women who gave birth less than a year and a half ago) during 2023 in Spain. We excluded women who had difficulty understanding the language (Spanish) and those under 18 years of age.

A random sample of 695 women in the perinatal stage would have to be drawn. This sample size has been calculated taking into account the following assumptions: 95% confidence level, 2% absolute error of precision, based on the population prevalence of suicidal ideation of 7% (Dudeney et al., 2023) and a non-response rate of 10%.

The form was distributed in health centers providing care and attention to pregnancy, childbirth and puerperium with the collaboration of nurses, physicians and midwives, among others. In order to answer possible questions and with the aim of giving the same treatment to every patient involved, a WhatsApp group was implemented so that the collaborating healthcare personnel could raise any doubts and concerns so that everyone would be aware of them.

### 2.1 Subjects and dataset

The information was collected through a self-developed questionnaire that had been piloted on a small group of women that included women of all socio demographic, educational and cultural characteristics. This questionnaire was written so that it could be understood by all women regardless of their education; It included both open and closed questions to gather information on the woman’s health, obstetric, pregnancy, personal and family history, as well as on lifestyles and toxic habits, age, level of education and income. Data on the newborn were also collected.

For each woman, pregnancy and childbirth were classified as low or high risk according to the criteria established in the health care providers’ pregnancy, childbirth and puerperium follow-up care plan. The risk of suicidal ideation was determined by the Paykel Scale, which was developed to assess the presence of suicidal thoughts (Martínez-Galiano et al., 2024).

This instrument specifically examines ideation related to death as well as suicide attempts. The scale is composed of five items, each of which uses a dichotomous yes/no response system with a score of 1 or 0. Scores range from 0 to 5 points, with questions referring to the past year. As scores increase, the frequency and severity of suicidal ideation also increase. For the purposes of this study, two cutoffs have been determined: one that is more sensitive, considering any positive response across the five items; and another that is more specific, determining a positive result based on affirmative answers to items 3, 4, and 5.

The Paykel Scale has been validated with good psychometric properties in a population similar to the study population. Descriptive statistics were used to analyze the data to describe the characteristics of the sample and calculate the prevalence of suicidal ideation risk.

The study obtained a favorable opinion to be carried out by the Jaén Provincial Research Ethics Committee (VAPAYPE-23-002). All involved women signed informed consent.

In order to ensure the best possible results from the model, some pre-processing techniques were applied on the dataset that contained the answers to the questionnaire. Since there were a large number of examples, those that were missing key information were removed so that the lack of data would not negatively impact the results of the predictive model. Other missing values were replaced by the average or mode when they could be computed.

Furthermore, all open-answer questions were discretized and turned into a series of broad-style answers to make the comparison and analysis of data easier. Lastly, values that involved dates, such as the date of the last delivery, were changed to the amount of years that had passed since then in order to simplify the training of the models.

As a result, the dataset included a total of 80 variables to analyze, which included whether the woman had had problems last year, her job, illnesses, techniques that were used during birth, the amount of cigarettes that the woman smoked prior to birth, the amount of visits to the hospital during pregnancy, satisfaction with birth, problems of intimate partner violence, the use of exclusive breastfeeding for the newborn, prior mental health assessments, age, weight of the newborn, alcohol consumption, years since the last birth, economic earnings, prior abortions, pain during birth, among others.

As the dataset was severely unbalanced, with only a small percentage of women showing a high risk of suicidal ideation, under sampling techniques were applied, creating several subsamples of the majority class to experiment with each of them to prevent overfitting.

Lastly, since the amount of examples was significant in spite of prior changes, the strategy used for training the models was that of 10-fold stratified cross validation, so that the modified dataset was divided in 10 subsets, called folds, that each had an individual response value akin to that of the original dataset. Each model is trained with all but one-fold, which is used to test its accuracy. This process is repeated 10 times, using a different fold to test the model’s results each time.

### 2.2 Classification models

In order to solve the classification problem, several models were used in order to compare the results and discover which was most appropriate for the problem at hand. Though many models exist, this research focuses on models that are both easy to understand, providing rules or trees that can aid doctors when diagnosing patients, and offer highly accurate results. This section includes an explanation of all models used, as well as the relevant hyperparameters for each model. These hyperparameters were selected due to the providing the most accurate results for each model and were obtained by fine-tuning the models on the training and test datasets.

#### 2.2.1 OneR

One Rule (OneR) (Holte, 1993) is one of the simplest models. It attempts to predict the label of a given example by generating one rule. It is the natural evolution of the ZeroR model, which simply predicts the majority label. The ZeroR model achieves an accuracy of 0.556 by simply predicting the majority class (considering all previous preprocessing, including under sampling techniques). These results, however, are not valid, as the model simply considers the most common class and constantly predicts it.

OneR generates an output, that is, a rule, in two steps. At first, it analyzes all the attributes that each example may have, and creates a rule for each of them. Afterwards, it tests every single generated rule and keeps the one with the highest accuracy, which will be used to make all predictions.

In spite of its simplicity, OneR can achieve high results in some cases, particularly when the datasets contain information that heavily correlates to a given class.

For this specific research, OneR was tested out with a minimum bucket size of 6 for discretizing numerical values, and 4 decimal places were considered.

#### 2.2.2 JRIP

JRIP is the java implementation of the RIPPER (Cohen, 1995) algorithm, which generates a ruleset that is further used to make predictions with the remaining examples. The algorithm creates the ruleset in two stages:

- Building stage: this stage generates rules until the error rate increases beyond a given threshold or no examples remain. A rule is generated by using one attribute, and antecedents are added to it by following a greedy approach (Hühn and Hüllermeier, 2009; Wang, 2023) until the rule is perfect. Afterwards, rules are incrementally pruned to prevent overfitting.
- Optimization stage: after generating the original ruleset, several variants of each rule are generated and pruned by following the same process as the building stage. One variant is generated by adding more antecedents, and the other is generated from an empty rule. The minimum description length for each original rule and its variants is computed, and the rule that has the lowest description length is selected and added onto the final ruleset.

The result is a series of ordered rules that represent the general behavior of a dataset, allowing not only for predictions of items but also for experts to discern the behavior of the data that is handled.

In this research, the error threshold was 50% and two variant rules were generated during the optimization stage.

#### 2.2.3 FURIA

Fuzzy Unordered Rule Induction Algorithm (Hühn and Hüllermeier, 2009), or FURIA for short, is an algorithm for generating a ruleset that allows for the prediction of labels. It was designed as an extension of JRIP that makes use of fuzzy logic.

As such, rules lack specific values that need to be met for the antecedent to become relevant; instead, a rule can be triggered with values that are within a certain range by making use of a given confidence factor. It also includes an efficient rule stretching method to deal with uncovered examples and unordered rulesets.

All of these changes allow FURIA to improve the results of JRIP in certain problems, particularly those with a large number of attributes or that would require a large ruleset in order to achieve accurate predictions.

As FURIA is based on JRIP, the hyperparameters used for FURIA are akin to those used in JRIP. Furthermore, the fuzzy AND-operator was defined as the Product T-Norm.

#### 2.2.4 J48

J48 (Ihya et al., 2019) is a decision tree that originated from C4.5 (Quilan Ross, 1993). A J48 tree consists of several nodes which can lead to other nodes based on the value of certain attributes of the data. The lowest-level nodes, also called leaf nodes, instead return a given label as output. To determine the label of a new example, the generated tree must be followed based on the specific value of the variables of that example.

The tree is built through several iterations of the same algorithm, which takes place in three main stages:

- Stage 1: If all or almost all elements of a node are of the same class or label, such a node is considered a leaf node, and the algorithm stops for that node.
- Stage 2: Otherwise, for each element in the node, all attributes are considered and tested. The attribute that presents the largest gain in data is selected, and the node is split into several child nodes. Each child node is defined by threshold values of the selected attribute, so that items of the parent node are distributed accordingly between all child nodes.
- Stage 3: Once the entire tree has been built by repeating stages 1 and 2, it can be pruned (Venkatesan and Velmurugan, 2015) by removing some nodes, thus preventing overfitting and sometimes increasing accuracy.

J48 has been tested in many applications, and has proven to be able to deal with particular characteristics, lost or missing attribute estimations of the data and varying attribute costs (Saravanan and Gayathri, 2018).

The J48 tree used in this research had a confidence factor of 0.25 and a minimum of 2 objects per leaf node. It was also designed to not use the actual value of nodes when it comes to splitting them, instead using the value that achieved the highest accuracy. Lastly, during the pruning, subtree raising was considered as to not fully remove them from the resulting tree.

#### 2.2.5 Random Forest

The Random Forest model (Breiman, 2001) is an ensemble model, and as such, it combines several simpler tree-based models to compute a final result. What differentiates Random Forest from other ensemble algorithms is the fact that, for every tree that it generates, only a portion of the attributes is considered. Afterwards, the prediction of each individual tree is combined with the rest by using a weighted sum or a majority vote in order to predict the label for a given example.

Since each tree considers different attributes and the information is later combined, the algorithm becomes more robust, particularly when compared with the individual trees that make up the model. Moreover, this partially prevents overfitting, as well as several other common issues that arise as a consequence of a high number of attributes.

The Random Forest was generated by using J48 trees, and all previously applicable hyperparameters are still used here. Furthermore, the model was tested by using several different amounts of trees. The best results were achieved by using 30 trees, which are the ones that will be considered.

### 2.3 Metrics

As accuracy may not be sufficient to determine the quality of a model that deals with the diagnosis of suicidal ideation risk, several metrics were used to compare the models to one another. To understand these metrics, a series of basic definitions upon which they are based will be presented:

- True Positive (TP): The amount of women with high suicidal ideation risk that are labeled as such.
- True Negative (TN): The amount of women without high suicidal ideation risk that are labeled as such.
- False Positive (FP): The amount of women without high suicidal ideation risk that are labeled as having a high suicidal ideation risk.
- False Negative (FN): The amount of women with a high suicidal ideation risk that are labeled as being low risk.

With these definitions, we can define the following metrics, which will be used to measure the performance of our models:

- Accuracy: (*TP* + *TN*)*/*(*TP* + *FP* + *TN* + *FN*)
- Precision: *TP/*(*TP* + *FP*)
- Recall: *TP/*(*TP* + *FN*)
  - F1 Score: 2 *×* (*Precision* × *Recall*)*/*(*Precision* + *Recall*)
  - AUPRC: (*Recall_n_* − *Recall_n-1_*) × *Precision_n_*

## 3 Results

This section includes general information about the sample used for research as well as the general results of all used models, present in section 3.1.

A total of 908 women took part in the study. The mean age of the women in the study was 31.34 with an standard deviation of 5.78, 90% (817) had planned the pregnancy, 85% (772) had worked during pregnancy, 79.7% (724) were healthy, without any pathology, 72.9% (662) had had a low obstetric risk pregnancy, 17.8% (162) had to seek health care to become pregnant, 56.2% (510) had a history of mental health disorders, for 51.7% (469) of the women this was their first child, 59.3% (538) stated that they had no religious beliefs whatsoever. A total of 60.2% (547) stated that they never drank alcohol during the perinatal stage. Applying the Paykel scale, 19.3% (175) of the women were identified as being at risk of suicidal ideation.

### 3.1 Results of the models

This section includes the general results of all tested models on Table 1.

**Table 1:** Performance metrics of all tested models.

Model	Accuracy (%)	Precision	Recall	F1	AUPRC
OneR	0.486 ± 0.02	0.487	0.486	0.486	0.484
JRIP	0.583 ± 0.055	0.583	0.583	0.583	0.583
FURIA	0.581 ± 0.045	0.577	0.581	0.579	0.577
J48	0.567 ± 0.1	0.565	0.567	0.566	0.561
Random Forest	0.639 ± 0.05	0.634	0.634	0.634	0.632

As shown, all models show lackluster results, with around 60% accuracy in most cases. The model that offers the worst results is OneR, which offers below 50% accuracy.

However, Random Forest achieves better results in every metric, though its results are still only about 60% accuracy. This implies that even if its results are better than those of every other model, they continue to not be enough for the importance of the task being handled. Regardless, these results continue to show a meaningful gain over baseline models such as ZeroR. Not only is the accuracy considerably superior, with roughly 10% more correctly-predicted results, but minority classes are additionally considered.

Nevertheless, all models can provide information on which sociodemographic and clinical data are relevant to determine whether a woman has a high risk of suicidal ideation. Further sections will explain the results of each model in detail.

#### 3.1.1 OneR

OneR achieved the worst accuracy of all models with just 48.6% accuracy, below that of ZeroR. Its model showed that the most relevant parameter was the weight of the baby after birth, with babies whose weight was below 2,910 g being associated with a higher suicidal ideation risk. The specific results are shown on Table 2.

**Table 2:** Rule generated by OneR.

Condition	Premise	Consequent	Prediction	Precision	Recall
IF	newborn_weight < 2,910	THEN	suicidal_ideation_risk = HIGH	0.437	0.450
ELSE	suicidal_ideation_risk = LOW			0.530	0.516

#### 3.1.2 JRIP

JRIP achieved a 58.3% accuracy. The rules that it generated imply that one of the most important factors to determine if a woman has a high suicidal ideation risk is whether she had already been assessed by mental health professionals prior to pregnancy. The weight of the child is also considered, following the exact same threshold value as discussed in OneR: 2,910 g. The specific rules generated by this algorithm appear on Table 3.

**Table 3:** Rules generated by JRIP.

Condition	Premise	Consequent	Prediction	Precision	Recall
IF	mental_health_assessment = true AND exclusive_breastfeeding = FALSE	THEN	suicidal_ideation_risk = HIGH	0.819	0.289
IF	daily_ciggarettes > = 1 AND newborn_weight <= 2,910	THEN	suicidal_ideation_risk = HIGH	0.813	0.203
IF	mental_health_assesment = true AND childbirth_satisfaction = somewhat	THEN	suicidal_ideation_risk = HIGH	0.731	0.369
ELSE	suicidal_ideation_risk = LOW			0.617	0.617

#### 3.1.3 FURIA

FURIA achieved an accuracy of 58.1%. The rules it generated applied fuzzy logic and a confidence factor (CF) to determine how likely a predicted diagnosis was based on specific values. For each attribute, the entire range of values was divided evenly across 5 labels: very low, low, medium, high and very high. Each label is defined by four values; if the example value is between the first and second values or between the third and fourth values, the chosen label is not certain, and the CF is considered. If no other labels are more likely, that label is used. If it is between the second and third values, the label is certain.

FURIA included more rules than any other previous model, and shares some rules with JRIP, implying that being assessed by mental health professionals prior to pregnancy is related to a high suicidal ideation risk. Moreover, it also considers that if a woman has problems of intimate partner violence and economic issues, the risk of suicidal ideation can increase.

Lastly, there also seems to be some correlation between smoking cigarettes prior to pregnancy, as women who smoked a lot (12+ cigarettes a day before pregnancy) tend to have a high suicidal ideation risk. The specific results are shown in Table 4.

**Table 4:** Rules generated by FURIA.

Condition	Premise	Consequent	Prediction	Precision	Recall
IF	mental_health_assessment = true AND exclusive_breastfeeding = FALSE	THEN	suicidal_ideation_risk = HIGH (CF = 0.77)	0.819	0.289
IF	mental_health_assessment = true AND maternity_leave IN [28, 29, 32, 33]	THEN	suicidal_ideation_risk = HIGH (CF = 0.8)	0.643	0.300
IF	problems_of_intimate_partner_violence = somewhat AND contract = freelance	THEN	suicidal_ideation_risk = HIGH (CF = 0.76)	0.611	0.285
IF	problems_of_intimate_partner_violence = somewhat AND childbirth_satisfaction = somewhat AND age IN [−inf, −inf, 38,39] AND sought_pregnancy = true	THEN	suicidal_ideation_risk = HIGH (CF = 0.78)	0.627	0.293
IF	daily_ciggarettes IN [11, 12, inf, inf]	THEN	suicidal_ideation_risk = HIGH (CF = 0.77)	0.619	0.289
IF	years_since_last_childbirth IN [5, 6, inf, inf] AND days_in_hospital IN [1, 2, inf, inf]	THEN	suicidal_ideation_risk = HIGH (CF = 0.9)	0.724	0.338
ELSE	suicidal_ideation_risk = LOW			0.605	0.668

#### 3.1.4 J48

J48 achieved an accuracy of 56.7%. Unlike the previous algorithms, J48 generates a tree that must be followed to reach a prediction. The tree can be easily turned into a ruleset, which is provided in Table 5.

**Table 5:** Rules generated by J48.

Condition	Premise	Consequent	Prediction	Precision	Recall
IF	daily_ciggarettes = 0 AND problems_of_intimate_partner_violence = high AND issues_last_year = true	THEN	suicidal_ideation_risk = HIGH	0.525	0.494
IF	daily_ciggarettes > = 1 AND issues_last_year = true	THEN	suicidal_ideation_risk = HIGH	0.509	0.472
IF	daily_ciggarettes > = 1 AND issues_last_year = false AND alcohol_consumption = frequent	THEN	suicidal_ideation_risk = HIGH	0.565	0.071
ELSE	suicidal_ideation_risk = LOW			0.598	0.628

This tree includes several of the same values that were previously mentioned, including the amount of cigarettes smoked prior to pregnancy and problems of intimate partner violence.

#### 3.1.5 Random Forest

Random Forest created a total of 30 J48 trees in order to analyze data and predict the suicidal ideation risk of women. By using these 30 trees, it achieved an accuracy of 63.9%. Some of the most relevant rules that were generated are shown in Table 6.

**Table 6:** Rules generated by Random Forest.

Condition	Premise	Consequent	Prediction	Precision	Recall
IF	mental_health_assessment = true AND exclusive_breastfeeding = FALSE	THEN	suicidal_ideation_risk = HIGH	0.819	0.289
IF	problems_of_intimate_partner_violence = somewhat OR single_mother AND issues_last_year = TRUE	THEN	suicidal_ideation_risk = HIGH	0.762	0.227
IF	daily_ciggarettes > = 8.5	THEN	suicidal_ideation_risk = HIGH	0.813	0.203
IF	monthly_income <1,000 AND exclusive_breastfeeding = FALSE	THEN	suicidal_ideation_risk = HIGH	0.772	0.143
IF	mental_health_assesment = true AND childbirth_satisfaction = somewhat	THEN	suicidal_ideation_risk = HIGH	0.731	0.369
ELSE	suicidal_ideation_risk = LOW			0.634	0.776

**Figure 1:**
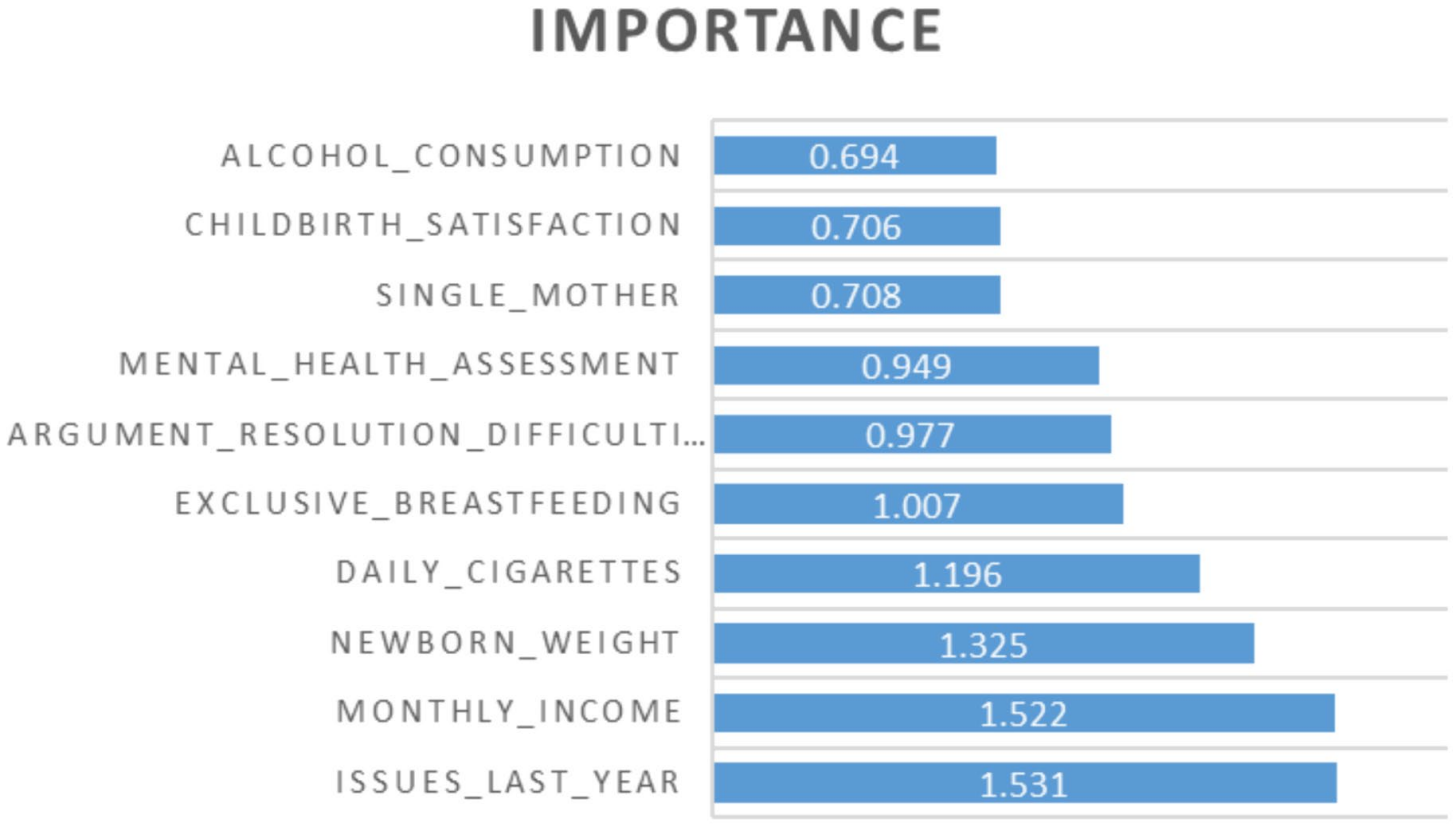
Top 10 variables considered by the Random Forest model.

Along with previously mentioned variables, such as problems of intimate partner violence or smoking, Random Forest also considers the monthly economic earnings of the woman’s household, with a lower earning correlating with a higher chance for suicidal ideation. The 10 most relevant variables considered by the Random Forest model are present in Figure 1. The importance of each variable was computed by considering the amount of times the variable was considered when building a tree, dividing the splits with its candidate and summing them up for each level of the tree.

It should also be noted that the recall for the minority class is of roughly 0.492. As such, only around half of the high-risk cases can be properly classified by the model. The model could potentially be changed and trained with oversampled cases of high-risk women to allow for specific fine-tuning to attempt to improve this score, as a false negative is the most dangerous error for this specific problem. Furthermore, alongside with these specific techniques, the overall limited results of all models show that they are not to be used as-is as the only method of detection, but more so as a supplement to medical professional’s analysis of women in potential risk. However, this continues to be an improvement over baseline models such as ZeroR, which achieves a recall score of 0% on the minority class. Even if only roughly half of the members of the minority class are correctly predicted, this continues to improve results of baseline models.

## 4 Discussion

The predictive models obtained for the risk of suicidal ideation in the perinatal stage based on sociodemographic, lifestyle and clinical-care data showed a mediocre accuracy of around 60%. In most of the models, the predictor variables that were repeated as the most important determinants were low birth weight of the newborn, a history of mental pathology in the woman, problems of intimate partner violence, low income and smoking.

Among the limitations of the study, it should be taken into account that it is an observational study based on surveys of women, so that a possible amnestic, anamnestic and non-response bias cannot be completely ruled out; however, given the characteristics of the information collected, as well as the time at which it was done, *a priori*, there are no elements that would lead one to think that the existence of any of these possible biases would have had a notable influence on the results.

As already mentioned, a multicausal origin in the risk of suicidal ideation is proposed, as with these predictive models there are a series of factors that have not been identified or included that make it impossible to predict approximately 40% of the cases. Among these possible causes, a genetic predisposition (Docherty et al., 2023), the influence of certain substances found in the environment (Kim et al., 2025) or the type of diet and food consumption have been identified (Lu et al., 2024; Tae and Chae, 2025).

The weight of the newborn, identified as a primary variable in the pre dictive models of suicidal ideation risk in the perinatal stage, is frequently considered a problem by the mother because of the consequences that it may have for the health and development of the newborn, although studies have been found in the literature that also associate low suicidal ideation risk in the perinatal stage with low birth weight (Gentile, 2011), as identified in our model. On the other hand, there is consolidated evidence regarding the association between the presence of alterations in the woman’s mental health and the risk of suicidal ideation (Anbesaw et al., 2021; Al-Sammak and Al-hamdany, 2025), as is also established in our predictive models as a determining factor. Similarly, the existence of conflict in the couple or the existence of problems of intimate partner violence emerged as an important predictor variable in some of the models in line with what was identified by different authors (Martínez-Vázquez et al., 2024; Al-Sammak and Al-hamdany, 2025).

A lower level of income may condition some of the actions, decisions or way of acting of the woman and may imply a limitation to being able to access the necessary resources necessary to live. The arrival of a new member to the family unit could further reduce this economic capacity which becomes another problem. Numerous authors, in accordance with the findings of the predictive models, identify the level of income as a variable associated with a higher risk of suicide (García-Martín et al., 2020; Cammack et al., 2024) although there are some who claim that there are not enough studies to establish this relationship (Barrass et al., 2024).

Finally, smoking has been associated with an increased likelihood of suicidal ideation, as found in the predictive models that have been developed. This toxic habit has been identified as a factor that increases the risk of suicidal ideation in women during pregnancy by other authors (Gelabert et al., 2024).

As it has been already mentioned, however, the models lack full understanding on the causes of suicidal ideation. As it mainly focuses on factors that are agreed upon as important by literature, there is a high chance that it lacks key information with which it could increase its ability to correctly make predictions.

Some studies (Adekkanattu et al., 2024) focus on the usage of Deep Learning techniques, achieving more accurate results (over 80% accuracy in most cases), at the cost of being black-box models that, in spite of the higher accuracy they provide, offer no information on the reasoning behind their predictions. As such, they are less desirable for a task as sensitive as the assessment of suicidal ideation in women.

Several studies focus on the use of AI techniques to determine possible causes for suicidal ideation, such as in Pigoni et al. (2024), where most of the studies achieve an accuracy of above 70%, which is somewhat better than our model. However, these studies also focus on a wide variety of patients and mental disorders beyond those of perinatal-stage women. This wider variety of patients, as well as the inclusion of biomarker data as well as sociodemographic and clinical data causes results to improve, leading to the belief that, if the presented models were to be trained with varied attributes beyond the ones that were already taken into consideration, higher accuracy would be achieved.

Due to this, it is likely that a wider variety of attributes that included biomarkers might make it possible to achieve higher accuracy while also keeping the advantages of machine learning over deep learning, providing information on the causes of a higher suicidal ideation risk. Our models, although with lesser predictive capacity, as they are based only on sociodemographic and clinical variables, may be more feasible to apply in the health system and detect the risk of suicidal ideation more quickly, without the need to obtain biological samples that may be painful and invasive for the woman and having to wait for the results of these.

## 5 Conclusion

The developed predictive models to determine the risk of suicidal ideation in the perinatal stage based on sociodemographic, clinical-care and lifestyle variables have a mediocre accuracy. The most influential predictors in these models are low birth weight, maternal smoking, low income and a history of mental health problems in the woman.

## 6 Relevance for clinical practice

This study highlights the potential for AI-based predictive models as a tool to identify perinatal women at risk of suicidal ideation. While the models’ accuracy is moderate, they can assist healthcare providers in early screening by flagging patients with risk factors such as low birth weight, mental health history, relationship conflicts, low income, and smoking. This facilitates timely intervention and support, recognizing that clinical judgment remains essential for the individualized assessment and care of patients. Therefore, it is recommended that suicidal ideation screening be performed during one of the prenatal appointments a woman attends in each trimester of pregnancy, the specific appointment to be determined by each healthcare system based on its organization and efficiency. It is also essential to perform this early screening during the postpartum visit and even during one of the newborn check-ups after the puerperium. All of this, as indicated, should be established according to the organization of each system and always supported by other tools and the supervision, assistance, intervention, and decision-making of a specialist healthcare professional. Currently, there is no screening established to detect the risk of suicidal ideation in prenatal and postnatal control. The model can be the basis and the beginning of this screening to detect those women at risk and subsequently clinical interviews can be carried out with mental health professionals. Likewise, if the professional, even though the model does not detect risk, but identifies elements that are compatible with the risk of suicidal ideation, this is the one that should prevail. As mentioned above, it is a support element that must be ratified with the clinical criteria of specialist health professionals.

## Data Availability

The data analyzed in this study is subject to the following licenses/restrictions: request for author corresponding. Requests to access these datasets should be directed to rpmolina@ujaen.es.
